# Therapeutic use of medical *Cannabis* in neurological diseases: a clinical update

**DOI:** 10.1007/s00702-023-02719-1

**Published:** 2023-11-28

**Authors:** Ute Hidding, Tina Mainka, Carsten Buhmann

**Affiliations:** 1 Department of Neurology, University Clinic Hamburg-Eppendorf, Martinistraße 52, 20246 Hamburg, Germany; 2 Department of Neurology and Experimental Neurology at the Charité, Charitéplatz 1, 10117 Berlin, Germany; 3 Berlin Institute of Health at Charité-BIH Biomedical Innovation Academy, BIH Charité Clinician Scientist Program, Charitéplatz 1, 10117 Berlin, Germany

**Keywords:** *Cannabis*, Cannabinoids, THC, Neurological diseases, Neurology

## Abstract

The use of medical *Cannabis* has increased in recent years due to changing legal circumstances in many countries. Approval exists only for a few neurological conditions such as rare forms of epilepsy or spasticity in multiple sclerosis. Beyond that, however, medical Cannabis is used for a wide range of neurological conditions and symptoms. In Germany, in parallel with new legislation that has simplified the prescription of medical *Cannabis*, an accompanying survey has been implemented for which initial data are now available. In this context, our review provides an overview of the evidence for the therapeutic use of medical *Cannabis* in neurology, the potential benefits, and side effects.

## Introduction

*Cannabis sativa and Cannabis indica* plants have been used for medicinal purposes for thousands of years (Pisanti and Bifulco 2017). The plants contain numerous substances, such as terpenes, flavonoids, phytosterols and phytocannabinoids, with tetrahydrocannabinol (THC) and cannabidiol (CBD) being of the greatest pharmacological importance. Since THC, unlike CBD, has a psychoactive effect, only the prescription of THC and its analogs falls under the Narcotics Law. THC and CBD enable their effect like endogenous cannabinoids (e.g., anandamide) via the G protein-coupled cannabinoid receptors CB1 and CB2 (Cristino et al. 2020). CB1 receptors are mainly localized in the central nervous system (e.g., basal ganglia, limbic system, spinal cord) and in the cardiovascular system. CB2 receptors are particularly found on immunoregulatory and hematopoietic cells and to a lesser extent in the central nervous system (Fife et al. 2015; Kluger et al. 2015; Cristino et al. 2020). In addition to CB1 and CB2 receptors, (endo)cannabinoids can interact with TRPV or GABA receptors, among others (Cristino et al. 2020). The endocannabinoid system is pharmacologically complex, its modes of action and interaction pathways have not yet been fully elucidated.

This could be one of the reasons why the application of *Cannabis* as medicine remains controversial. In recent years, access to medical Cannabis has been facilitated in many countries. In Germany, patients with a serious illness can be prescribed *Cannabis* flowers and extracts or synthetic cannabinoids at the expense of the health insurance companies since 2017, but the scientific basis for a meaningful prescription remains unclear for most indications due to a lack of studies. Remarkably, for the “not entirely remote prospect of a noticeable positive impact on serious symptoms” required by the legislature in the treatment with medicinal *Cannabis* (Bundestag 2017), randomized and controlled studies are not necessary. On the other hand, the current evidence allows a reasonable use only in rare cases (Montero-Oleas et al. 2020; Mainka and Buhmann 2023) (see Table 1). Furthermore, suitable therapies do not have to be available for treatment or these “[cannot] be used after weighing the expected side effects and taking into account the medical condition of the insured person” (Bundestag 2017). This can be the case, for example, in the case of tolerability problems with an established therapy.

**Table 1:** Evidence for efficacy and state of approval in Germany for cannabinoids in neurological diseases

Indication	Evidence for efficacy	State of approval
Epilepsia		
Lennox Gastaut syndrome	+	Epidyolex^®^ (Cannabidiol) as add-on to Clobazam from the age of 2 years
Dravet syndrome	+	Epidyolex^®^ (Cannabidiol) as add-on to Clobazam from the age of 2 years
Tuberous sclerosis	+	Epidyolex^®^ (Cannabidiol) from the age of 2 years
Pharmacotherapy-resistant epilepsy syndromes	(+)	n/a
Multiple sclerosis	+	Sativex^®^ as add-on for moderate to severe spasticity
Pain		
Neuropathic pain syndromes	(+)	n/a
Headache syndromes	?	n/a
Gilles de la Tourette syndrome	(+)	n/a
Neurodegenerative diseases		
Parkinson’s disease	(+)	n/a
Huntington’s disease	?	n/a
ALS	?	n/a
Dementia	?	n/a
Dystonia	?	n/a

Classification of efficacy according to current scientific knowledge: “+” effective, "(+)" possibly effective, “−“: ineffective, “?”: questionable, n/a: no approved drug available

Since the introduction of the German law “Cannabis as Medicine” in 2017, data on the use of *Cannabis* medicinal products has been collected in an accompanying survey by the Federal Institute for Drugs and Medical Devices (Bundesinstitut für Arzneimittel und Medizinprodukte [BfArM]), which was published in July 2022 (BfArM 2022a). In addition to an overview of the evidence of cannabinoids in possible treatment indications in the spectrum of neurological and neuropsychiatric diseases, this article also examines the findings of the statistical accompanying survey over the first 5 years “cannabis on prescription” in the neurological field.

## Neurological diseases

### Epilepsia

For several years, cannabidiol (Epidiolex^®^/Epidyolex^®^) has been approved in the US and in Europe as an add-on therapy for the treatment of two forms of epilepsy, which are often drug-resistant, Lennox–Gastaut (LGS) and Dravet syndrome (Thiele et al. 2018; Stockings et al. 2018). It was also recently approved for seizures associated with tuberous sclerosis (Thiele et al. 2021, 2022). In addition to these rare epilepsy syndromes, meta-analyses and systematic reviews also show the effectiveness of CBD for other difficult-to-treat epilepsy syndromes (Stockings et al. 2018; Elliott et al. 2019; Lattanzi et al. 2021). The most frequently reported undesirable side effects in the treatment of mostly young epilepsy patients were diarrhea, somnolence, fever, decreased appetite, and vomiting (Devinsky et al. 2017; Thiele et al. 2018). In addition, patients take cannabinoids as self-medication: a survey in the USA showed that they achieve significantly better seizure control with daily use (Kerr et al. 2019). However, more precise data are missing. According to the German survey, epilepsy (ICD-10 G40.-) was the reason for the prescription of medical Cannabis in only a few cases (< 1%). Though this survey only includes preparations containing THC, so pure CBD preparations are not recorded (BfArM 2022b).

In the case of pharmacotherapy-resistant epilepsy syndromes for which *Cannabis* preparations have not yet been approved, experts consider a therapy attempt with highly purified or synthetic CBD when guideline-compliant medication is not sufficient (Potschka and Brandl 2017). To monitor therapy, it is advisable to document the frequency and severity of seizures before and during therapy.

### Multiple sclerosis

According to a survey in Canada, self-medication with THC-containing cannabinoids appears to be widespread among patients with multiple sclerosis (MS). Target symptoms are sleep disturbances, pain, and spasticity, with the most commonly reported side effects being drowsiness, emotional flattening, and difficulty concentrating (Santarossa et al. 2022). The oromucosal spray Sativex^®^ (nabiximols) contains equal quantities of THC and CBD. It is already approved in Canada and most European countries for the treatment of moderate to severe spasticity in MS (Wade et al. 2010; Markovà et al. 2019). However, the possible benefit of the drug does not seem to have been fully exhausted due to partially unclear guidelines and recommendations (Carod-Artal et al. 2022). Sativex^®^ and other cannabinoids are well tolerated by patients with MS. Also, taking Sativex^®^ should not have a negative effect on the ability to drive of MS patients (Freidel et al. 2015).

In addition to spasticity, effects of medicinal *Cannabis* on pain (Russo et al. 2016) and neurogenic bladder dysfunction (Maniscalco et al. 2018) in MS have been reported although systematic reviews and meta-analyses suggest only a limited effect of cannabinoids (Nielsen et al. 2018; Torres-Moreno et al. 2018). Possible neuroprotective effects of medical *Cannabis* are also suggested in MS (Gado et al. 2018; Sorosina et al. 2018).

Overall, 5.9% of the patients in the German survey were prescribed cannabinoids due to MS (ICD-10 G35.-). Dronabinol (44.4%) was mostly prescribed, followed by *Cannabis* flowers (35%) and Sativex^®^ (16.2%) (BfArM 2022a, b).

### Pain

Although numerous studies with cannabinoids have already been executed in the broad field of pain, a carefully carried-out systematic review found only low-level evidence of pain reduction (Fisher et al. 2021). A Cochrane review of *Cannabis*-based medicine for chronic neuropathic pain, calculated the “number needed to treat” for an additional benefit (pain reduction of at least 50%) as 20. In contrast, the “number needed to harm” was set at 25; however, the level of evidence was low (Mücke et al. 2018). Nevertheless, the authors concluded from the data that the potential benefit of *Cannabis*-based medicine in chronic neuropathic pain outweighs the potential risks (Mücke et al. 2018). Cannabinoids are also often used in self-medication for headache syndromes such as migraine. In migraine, cannabinoids for the treatment of attacks must be distinguished from its use for prophylaxis. Cannabinoids are reported to reduce both frequency and duration of attacks (Poudel et al. 2021). However, high-quality RCTs are also missing (Patel 2021), so that the need to collect reliable data on cannabinoids in pain medicine is still very high, particularly for conditions for which effective evidence-based therapies are available, such as topiramate or GCRP antagonists for migraine prophylaxis (Haroutounian et al. 2021).

Despite the insufficient scientific evidence in this field, according to the German survey of the BfArM, pain syndromes were the most common reason for the prescription of cannabinoids with 76.4% of the cases (BfArM 2022a). Dronabinol was the drug prescribed most for both chronic pain (ICD-10 R52.1, R52.2) and neuropathic pain (ICD-10 G95.85, G50-G64, M79.2) (62.2% and 67%, respectively) (BfArM 2022b). In addition, cannabinoids were also prescribed for separately recorded headache syndromes such as migraine (2%), cluster headache (0.6%) or trigeminal neuralgia (0.9%) (BfArM 2022a).

While dronabinol was also primarily prescribed for migraine (ICD-10 G43.-) and trigeminal neuralgia (ICD-10 G50.-) (49.7% and 63.3% respectively), *Cannabis* flowers (35.4%) were the most frequently prescribed substance for cluster headaches (ICD-10 G44.0) (BfArM 2022a). According to the BfArM, the quality of life for the indication pain improved moderately (36.6%) or even significantly (32.7%) with the use of cannabinoids in most cases (BfArM 2022a). These data contradict those from a meta-analysis that did not show any improvement in quality of life from cannabinoids in neurological (and oncological) diseases (Belgers et al. 2023). The most commonly reported side effects in pain patients were fatigue (16.3%), dizziness (12.5%) and nausea (7.4%) (BfArM 2022a). In patients whose therapy with cannabinoids was discontinued, one-third did so because of side effects (31.2%) or even more frequently because of ineffectiveness (44.8%) (BfArM 2022a).

### Tic disorders

Cannabis-based medicine has been increasingly used for primary tic disorders such as Gilles de la Tourette syndrome (GTS) since the first positive reports in the 1980s and 1990s (Sandyk and Awerbuch 1988; Müller-Vahl et al. 1999). According to a survey by the European Society for the Study of GTS, THC was already the 7th most common drug prescribed to treat motor and vocal tics (Roessner et al. 2022).

The prescribing practice seems contrary to the evidence available so far. A meta-analysis—albeit only based on two small RCTs (Müller-Vahl et al. 2002, 2003)—could not demonstrate a significant effect of THC on tics in GTS (Black et al. 2019). Not only the administration of THC, but also the combination with CBD, such as with Sativex^®^, has already been explored in GTS. The application seems to be safe; however, there are only retrospective evaluations, case reports and open uncontrolled studies, that do not prove an effect considering the number of possible preparations, application forms and dosages (Trainor et al. 2016; Kanaan et al. 2017; Milosev et al. 2019; Anis et al. 2022).

Another attempt to treat primary tic disorders with *Cannabis*-based medicine is monoacylglycerol lipase (MAGL) inhibition, which is said to prevent the breakdown of endocannabinoids (Pan et al. 2009). Although this approach showed a positive effect on the severity of symptoms in GTS in a phase Ib study (Artukoglu and Bloch 2019), it could not be confirmed in a phase II study (Müller-Vahl et al. 2021).

Despite the lack of many high-quality studies, experts recommend *Cannabis* preparations as second-line treatment of patients with tic disorders who are otherwise refractory to drug and behavioral therapy (Ludolph et al. 2012; Müller-Vahl 2013; Roessner et al. 2022).

According to the German survey, 0.6% of the patients who were prescribed *Cannabis* on prescription had the diagnosis of GTS (ICD-10 F95.2). These patients were most frequently prescribed *Cannabis* flowers (42%), followed by Sativex^®^ (27%) and dronabinol (25%) (BfArM 2022b).

### Neurodegenerative diseases

#### M. Parkinson

The reported self-use of cannabinoids in patients with Parkinson's disease (PD) ranges from 8.3 to 37% (Venderová et al. 2004; Kindred et al. 2017; Feeney et al. 2021; Yenilmez et al. 2021; Erga et al. 2022). Cannabinoids are used to improve motor symptoms, such as tremor, bradykinesia, rigidity, levodopa-induced dyskinesia and freezing as well as non-motor symptoms such as pain, anxiety or sleep disorders (Venderová et al. 2004; Kindred et al. 2017; Feeney et al. 2021; Yenilmez et al. 2021; Erga et al. 2022). The inhalative use of THC-containing cannabinoids seems to be preferred by patients (Venderová et al. 2004; Kindred et al. 2017; Yenilmez et al. 2021). In addition to improving symptoms, taking cannabinoids can also reduce the dosage of the prescribed anti-parkinsonian medication (Kindred et al. 2017). However, many patients also stopped self-medication with cannabinoids due to ineffectiveness (Feeney et al. 2021). Side effects include dry mouth, dizziness, and cognitive changes (Holden et al. 2022).

Regarding motor symptoms, two meta-analyses examined the effect of medical *Cannabis* mostly measured on the motor part of the MDS-UPDRS (Movement Disorder Society-Unified Parkinson's Disease Rating Scale). Contrary to the reports of patients, they found no convincing evidence for the use of various *Cannabis* preparations in PD (Thanabalasingam et al. 2021; Urbi et al. 2022). However, these meta-analyses consist of only 15 studies including 6 randomized controlled studies (RCTs) (Thanabalasingam et al. 2021) and 18 studies including 5 RCTs (Urbi et al. 2022), respectively. Again, in addition to the heterogeneity of the type of application, the proportion of THC and/or CBD in the studies is heterogeneous (Frankel et al. 1990; Sieradzan et al. 2001; Carroll et al. 2004; Zuardi et al. 2009; Chagas et al. 2014) or even unknown (Lotan et al. 2014).

The placebo-controlled, double-blind “NMS-Nab Study” examined the effect of the synthetic THC-analog nabilone on non-motor symptoms in PD (Peball et al. 2019). Positive effects have been demonstrated for anxiety and sleep (Peball et al. 2020, 2022). The most common side effects, mostly mild in severity, were fatigue and drowsiness, dizziness, dry mouth and confusion (Peball et al. 2020). In a double-blind, placebo-controlled study, Parkinson's patients with REM sleep disorders did not benefit from CBD administration (Almeida et al. 2021). A post hoc analysis of the same study also showed no benefit in terms of additional restless legs symptoms (de Almeida et al. 2023).

According to the German survey of the BfArM, PD (ICD-10: G20.-) was the reason for the prescription of cannabinoids in only < 1% of cases, mostly dronabinol or Sativex^®^. Therefore, a more extensive analysis for PD is not possible from this evaluation (BfArM 2022b). In particular, it remains unclear whether there is an increased risk of hallucinations under medication with cannabinoids (Cravanas and Frei 2020).

In addition to the direct effects in the endocannabinoid system, CBD has also been attributed antioxidant and anti-inflammatory effects, which have led to a reduction in striatal neurodegeneration in Parkinson's animal models (Bhunia et al. 2022; Muhammad et al. 2022). However, translation to humans has not yet been attempted.

#### Huntington’s disease

The effect of CBD, nabilone and Sativex^®^ on motor and non-motor symptoms in Huntington's disease (HD) was examined in three crossover studies (Consroe et al. 1991; Curtis et al. 2009; López-Sendón Moreno et al. 2016). While no significant positive effect on motor symptoms like chorea could be demonstrated, nabilone led to an improvement in neuropsychiatric symptoms (Curtis et al. 2009). In an uncontrolled case series, the administration of Sativex^®^, dronabinol or nabilone in early-onset HD patients led to an improvement in motor skills, especially dystonia, as well as a reduction in neuropsychiatric symptoms (Saft et al. 2018).

Possible neuroprotective effects were examined more closely in rodent models of Huntington's disease, with indications of a slowdown in striatal degeneration and thus disease progression (Sagredo et al. 2011; Valdeolivas et al. 2012, 2017). However, this has not yet been reproduced in humans (López-Sendón Moreno et al. 2016).

#### Motor neuron disease

In motor neuron diseases like amyotrophic lateral sclerosis (ALS), cannabinoids have been studied for spasticity so far. A randomized, double-blind crossover study was unable to demonstrate a reduction in cramps after THC intake in ALS patients (Weber et al. 2010). However, Sativex^®^ decreased spasticity measured by the modified Ashworth scale in a double-blind, placebo-controlled phase II study (Riva et al. 2019). The most frequently recorded side effects were weakness, dizziness, fatigue and dry mouth (Riva et al. 2019). Despite this positive data, according to the German survey, cannabinoids were only prescribed in rare cases (< 1%) for motor neuron diseases (G12.2); most patients used dronabinol (BfArM 2022b).

In transgenic mice, CB1 and CB2 receptor agonists have already been able to slow disease progression, so the neuroprotective effect of a *Cannabis*-based extract with high CBD and low THC content is now to be tested in recently diagnosed ALS patients (Urbi et al. 2019).

#### Dementia

Only a few studies have examined the benefits of cannabinoids in dementia syndromes, specifically for the treatment of behavioral abnormalities (Volicer et al. 1997; Walther et al. 2006). Reliable data from RCTs do currently not exist.

There is evidence from animal experiments and in vitro studies that cannabinoids can reduce the hyperphosphorylation of tau protein and the production of beta-amyloid in Alzheimer's dementia (Karl et al. 2017). Furthermore, cannabinoids are said to be able to reduce neuroinflammatory processes and oxidative stress in dementia via activation of microglia (Karl et al. 2017; Talarico et al. 2019). Apart from these pathophysiologically interesting approaches, there is currently no scientific evidence for the use of cannabinoids in dementia syndromes (Brucki et al. 2021).

### Dystonia

So far, there is no reliable data on the treatment of idiopathic dystonia with cannabinoids (Mascia et al. 2020). Individual cases describe a positive effect of CBD up to 600 mg/day in patients with cervical dystonia, generalized dystonia or Meige syndrome (Consroe et al. 1986; Sandyk et al. 1986), as well as of dronabinol on blepharospasm (Gauter et al. 2004) and of THC in musician's dystonia (Jabush et al. 2004). A small, randomized, double-blind, crossover study in patients with cervical dystonia showed no effect of dronabinol versus placebo (Zadikoff et al. 2011). In another randomized, double-blind crossover study on a patient collective with heterogeneous dystonia diagnoses, a single dose of nabilone did not alleviate dystonic symptoms (Fox et al. 2002). In contrast, a small, controlled pilot study with THC-containing *Cannabis* oil showed a symptom reduction in patients with blepharospasm (Zloto et al. 2022). In addition, in a retrospective data analysis, the benefits of medical *Cannabis* for a few patients with blepharospasm could be proven (Radke et al. 2017).

## Long-term effects of cannabinoids

Cannabis used for recreational purposes is assumed to have long-term effects in addition to the well-known short-term effects. These effects can vary based on factors, such as frequency of use, THC content, age of the user and individual susceptibility. Chronic use can lead to cognitive decline in some domains, such as verbal learning, and memory and speed of processing (Bourque and Potvin 2021). Notably, younger and especially adolescent people are more likely to experience adverse cognitive effects than older people (Mueller et al. 2021) suggesting a lower cognitive risk in the predominantly older patients with neurodegenerative diseases, but not in the often younger patients with other conditions such as migraine, tics or epilepsy. Long-term effects also include psychiatric disorders such as psychosis, anxiety and depression or addiction (Hasbi et al. 2023). The database on long-term effects of medicinal *Cannabis* use is very weak. A meta-analysis in chronic non-cancer pain concluded that long-term use is probably safe, but the included studies had a maximum observation period of 12 months. (Bialas et al. 2022). Regarding the use of cannabinoids in MS, the Cochrane Society states that psychiatric disorders may be increased compared to placebo (Filippini et al. 2022) while an observational study even found positive effects on cognition in MS (Alessandria et al. 2020). Overall, the long-term safety of medical *Cannabis* is still unclear.

## Practical advice

In general, the dosing of *Cannabis* preparations should be started with a low dose and only increased slowly (“start low and go slow”). In the case of individual dosing, the different effective latency and the duration of action for different types of application must also be taken into account. When inhaled, the active substance is absorbed quickly, but has an overall shorter duration of action; when substances are taken orally, the onset of action is delayed, but the effect lasts longer. The use as a tea—cannabinoids are fat-soluble—or as baked goods (“edibles”) is not recommended for medical purposes due to the poor control of the ingested dose (Mainka et al. 2018; Kassenärztliche Bundesvereinigung 2023).

It is recommended that a patient ID card for medical *Cannabis* is handed over with the prescription. Such cards are usually based on the well-known opioid cards in terms of form and content and are available online and free of charge from companies or interest groups. Patients should carry this card with them together with a copy of their last prescription (Grotenhermen and Häußermann 2017). For traveling abroad (EU) taking along medical *Cannabis* is in general possible for the patient. For this, it is necessary to carry an EU-wide valid certificate, filled out by a doctor in accordance with Article 75 of the Schengen Convention. This document has to be certified before by the competent authority in the respective federal state. It is strictly recommended to check the entry/import conditions depending on the destination country before traveling (Grotenhermen and Häußermann 2017).

### Prescription of cannabinoids in Germany

Currently, any medical doctor in Germany can prescribe cannabinoids. The regulations on the prescription of narcotics only apply to the active substance THC. The entitlement to the supply of *Cannabis* applies according to §31 paragraph 6 SGB V, if “a generally recognized service according to the medical standard is not available or cannot be used in the individual case […] taking into account the side effects to be expected and taking into account the patient's medical condition” (Bundestag 2017). In addition, there must be “a not entirely remote prospect of a noticeable positive effect on the course of the disease or on serious symptoms” (Bundestag 2017). In order for the costs of cannabinoid therapy to be covered by the health insurance, the prescriber must obtain authorization from the insurance before the first prescription. This does not apply to the prescription of specific cannabinoids for the limited indications for which an approval exists. The health insurance fund has a processing time of three weeks (5 weeks if an expert opinion is necessary) and can only refuse the application in justified exceptional cases. Nevertheless, around 40% of the requests are rejected. These bureaucratic hurdles probably mean that not all patients who could benefit from cannabinoids are supplied with them.

## Historical overview and handling of *Cannabis* use in other countries and cultures

Despite the fact that *Cannabis* herbs have been used for recreational, medicinal and spiritual purposes in many cultures for thousands of years, today the use and trade of *Cannabis* is strictly regulated in most countries.

A broader medical application started in Europe, when the physician William B. O'Shaughnessy, stationed in India, successfully treated his patients with (Indian) hemp in 1839 and made these results known in the Western world. Within a few years, the *Cannabis* herb imported from India and the preparations made from it were able to establish themselves as valued medicines. Medical *Cannabis* was in use for about 100 years, but its importance declined again after the First World War. The second International Opium Convention decided in 1925 mainly for economic and political reasons that the (THC-containing) raw resin (charas), which is extracted from the female tops of the *Cannabis sativa*, and all various preparations of it should not been utilized for medical purposes. The ban on medical use was subsequently enforced at various times in different countries, for example with the Dangerous Drug Act of 1928 in Great Britain or the Marijuana Tax Act passed in 1937 in the USA, which was intended to eliminate the use of hemp at all levels. In Germany, *Cannabis* was banned in 1951 as medicine, enacted as part of the Narcotics Act, which severely restricted the medical use of *Cannabis*.

In recent decades, many countries have relaxed regulations and, in some cases, legalized *Cannabis* to a large extent. Apart from the Netherlands, Uruguay was a pioneer in this respect, allowing cultivation, distribution, and consumption as early as 2013. Meanwhile, *Cannabis* is also legal in many states in the USA and in Canada with varying regulations on cultivation, distribution, and consumption. A special case is India, where a drink called "bhang" has been made from hemp leaves for thousands of years and is widely used as part of the Hindu tradition. Nevertheless, medicinal and recreational *Cannabis* is currently illegal in India.

## Cannabinoids and driving

In general, driving after recreational use of THC is an administrative offense in most countries. However, the German legislature differentiates between consumption according to a medical prescription and illegal consumption. In principle, the rules for driving a motor vehicle apply as for other neurological patients (Buhmann and Gerloff 2013). If the ability to drive is impaired by the cannabinoids (as is also the case with other drugs with effect on the central nervous system), driving a car or bicycle is not permitted. If there are doubts about the fitness to drive, this must be clarified between the patient and the doctor.

The authors of this article propose the following verbal information and written documentation for the patients, without any legal obligation being derived from this: During the dosing phase of THC-containing cannabinoids, no motor vehicle should be driven until the long-term dose therapy has been found. If a stable dose has been found and there are no side effects in the form of falling asleep, severe tiredness or other symptoms that might impair driving ability, a motor vehicle may be driven. A copy of the medical certificate of the prescription should be kept in the motor vehicle so that it can be shown to the police if necessary.

## Conclusion and suggestions for daily clinical practice

So far, there has only been reliable evidence for the effectiveness of cannabinoids in neurology for a few indications. These primarily include the epilepsy syndromes Dravet and Lennox–Gastaut syndrome as well as seizures in tuberous sclerosis, for which CBD as Epidyolex^®^ is approved for treatment. There is also approval for Sativex^®^, an extract of equal parts THC and CBD, for the treatment of spasticity in multiple sclerosis.

In the case of neurodegenerative and neuroimmunological diseases such as multiple sclerosis, data from animal models and in vitro studies raise hope for neuroprotective effects of cannabinoids in humans as well, but the translation has not yet been successful.

Unfortunately, the findings from the German survey by the BfArM do not go far beyond descriptive data, primarily for methodological reasons, and therefore cannot provide any additional evidence for the use of *Cannabis* for medical purposes (Kurz and Lau 2022). Further high-quality studies are therefore urgently needed to create a valid databases.

In the case of some diseases, such as tic disorders and chronic pain disorders, cannabinoids are already regularly used for treatment—despite the limited scientific evidence to date—insofar as other therapy options are not effective or are not well tolerated. Based on the results of two large national patient surveys, the use of cannabinoids in Parkinson's disease can be particularly useful for therapy-resistant non-motor symptoms, such as pain, sleep disorders, RLS or anxiety, but also to improve motor symptoms. Since the tolerability of even THC-containing cannabinoids seems to be generally good, we suggest that patients with serious symptoms should not be denied access to this therapy option if other therapy approaches have been proven insufficient. Depending on the disease, it is advisable to use validated scales and symptom diaries to objectify the success of therapy.

## Data Availability

Not applicable.
